# Maternal high-fat diet prevents developmental programming by early-life stress

**DOI:** 10.1038/tp.2016.235

**Published:** 2016-11-29

**Authors:** M Rincel, A L Lépinay, P Delage, J Fioramonti, V S Théodorou, S Layé, M Darnaudéry

**Affiliations:** 1 INRA, Nutrition et Neurobiologie Intégrée, UMR1286, Bordeaux, France; 2 Université de Bordeaux, Nutrition et Neurobiologie Intégrée, UMR1286, Bordeaux, France; 3 INRA, Toxalim, UMR1331, Toulouse, France

## Abstract

Anxiety disorders and depression are well-documented in subjects exposed to adverse childhood events. Recently, maternal obesity and/or maternal consumption of high-fat diets (HFD) have been also proposed as risk factors for offspring mental health. Here using an animal model in rats, we explored the combinatorial effects of a maternal HFD (40% of energy from fat without impact on maternal weight; during gestation and lactation) and maternal separation (MS) in offspring. In the prefrontal cortex (PFC) of pups, MS led to changes in the expression of several genes such as *Bdnf* (brain derived neurotrophic factor), *5HT-r1a* (serotonin receptor 1a) and *Rest4* (neuron-restrictive silencer element, repressor element 1, silencing transcription factor (*Rest*), splicing variant 4). Surprisingly, perinatal HFD strongly attenuated the developmental alterations induced by MS. Furthermore, maternal HFD totally prevented the endophenotypes (anxiety, spatial memory, social behavior, hypothalamic–pituitary–adrenal (HPA) axis response to stress, hippocampal neurogenesis and visceral pain) associated with MS at adulthood. Finally, we also demonstrated that HFD intake reduced anxiety and enhanced maternal care in stressed dams. Overall, our data suggest that a HFD restricted to gestation and lactation, which did not lead to overweight in dams, had limited effects in unstressed offspring, highlighting the role of maternal obesity, rather than fat exposure *per se,* on brain vulnerability during development.

## Introduction

The etiology of the majority of psychiatric disorders remains unknown. It is, however, well-accepted that psychosocial adversity in childhood can contribute to an increased risk of depressive and anxiety disorders later in life.^1, 2, 3, 4, 5, 6^ In modern societies, a considerable amount of the population including childbearing women and children is exposed to low-cost energy-dense food with a high content in fat. Yet, it has been recently proposed that maternal obesity and/or maternal consumption of fat-rich diets could also constitute risk factors for offspring's mental health.^7^ It is therefore crucial to unravel the possible combinatorial effects of perinatal exposure to fat-rich diets and early-life stress on the developing brain.

Early disruption of the mother–infant relationship in rats leads to a wide range of abnormalities^8, 9^ that are also found in depressive and anxious patients with an history of early-life stress.^6^ These include altered hypothalamic–pituitary–adrenal (HPA) axis response to stress,^10, 11^ reduced hippocampal neurogenesis,^12^ altered emotionality,^11, 13^ increased visceral pain^14^ and cognitive impairments.^12, 15^ Similarly, beside the well-known effects on offspring metabolism,^16, 17^ maternal obesity and/or maternal high-fat diet (HFD) consumption can also affect behavior and brain function in offspring.^18^ Indeed, altered hippocampal neurogenesis,^19^ spatial learning deficits^20^ and hyperanxiety,^21, 22, 23^ have been reported, suggesting that maternal stress and maternal HFD may produce similar effects on the brain during development.

In humans, early-life adversity has marked impact on child brain and particularly on the prefrontal cortex (PFC).^24, 25^ In rodents, ontogenic molecular changes within the PFC (between post natal day (PND) 7 and PND14) have been described in pups submitted to maternal separation (MS) and are suggested to participate to the programming effects of early-life stress.^26, 27^ Indeed, the increase of the neuronal transcription factor *Rest4* (neuron-restrictive silencer element, repressor element 1, silencing transcription factor (*Rest*), splicing variant 4) in pups' medial prefrontal cortex (mPFC) is responsible for the molecular signature of MS, characterized by upregulation of genes such as *5HT-r1a* (serotonin receptor 1A) and *Bdnf* (brain derived neurotrophic factor).^27^ Moreover, *Rest4* overexpression in the mPFC specifically during early post-natal development, but not in adulthood, is sufficient to produce MS-associated adult endophenotypes, especially hyperanxiety. Here we aim to determine whether exposure to maternal HFD in rats can mimic MS and potentiate the MS-induced developmental alterations in the PFC. To dissociate HFD effect from maternal obesity effects, we used a protocol of maternal HFD exposure (40% from fat, restricted to gestation and lactation periods), which does not produce maternal obesity.^28^ We further aim to evaluate the long-lasting impact of maternal HFD exposure on MS-induced alterations of emotional and cognitive behaviours, as well as some typical neuroendocrine and neurobiological changes affected by MS. Since MS is widely used as an animal model of irritable bowel syndrome (IBS),^14^ we also examined the effects of these early manipulations on visceral pain in adulthood. Finally, given that the dams were directly exposed to the HFD, and that previous reports suggest that HFD could modulate stress response, we also examined the behavioral effects of HFD in stressed dams.

## Materials and methods

### Experimental procedures

All experiments were approved by the Bioethical committee of our University (N° 50120186-A) and région Aquitaine Veterinary Services (Direction Départementale de la Protection des Animaux, approval ID: A33-063-920) according to the European (Directive 2010/63/EU, 22 September 2010) legislation.

Animals were maintained in a 12-h light/12-h dark cycle (lights on at 0800 hours) in a temperature-controlled room (22 °C) with free access to food and water. Seventy six pregnant female Wistar rats (11-week old, Janvier, Le Genest, Saint-Isle, France) were randomly assigned to either standard diet (SD) or HFD. A mixture of vegetable oils was used as the source of fat (SD, 12% and HF, 40% of energy from fat, Supplementary Table 1).^28^ Dams were maintained under these diets from the first day of gestation to postpartum day (PP) 21. At birth, litters were culled to 8–10 pups with balanced sex-ratios and randomly assigned to control group or MS group. From PND2 to PND14, stressed pups underwent daily MS for 180 min as previously described.^29^ During the separation sessions, dams were placed in new cages with free access to food (according to their respective diet) and water, whereas pups were placed in individual containers in another room under controlled temperature (28±2 °C). Control pups remained undisturbed with the dams. At PND21, male pups were weaned onto laboratory chow and housed four per cage (from different litters) until the testing age (Supplementary Figure S1). A maximum of two pups per litter was used for each measure to prevent from any litter effect.^30^ To investigate the effects of maternal diet and MS on brain during development, the expression of genes (*Rest4*, *Rest*, *5HT-r1a*, *Bdnf*, *Adcy5*, *Camk2a* and *Crh*) known to be affected by early stress^26, 27^ was assessed in the PFC at PND11 (cohort 1, see gene list in Supplementary Table 2). For that purpose, male pups of the four groups (SD–control, HFD–control, SD–MS and HFD–MS) were killed on PND11, with stressed pups killed either before or after the 180 min-period of MS. PND11 time point was chosen based on previous studies showing ontogenic changes in gene expression between PND7 and PND14 in MS pups.^26, 27^ Plasma levels of metabolic hormones were also assessed after 180 min of separation at PND11. In adulthood (cohort 2), rats underwent a battery of behavioral tests. First, they were tested for anxiety-like behaviour (4 months), then for spatial learning, spatial memory (5 months), anhedonia (6 months) and social interaction (7 months). At 8 months, they were killed and a random subset of each group was used for biochemical or immunohistochemistry analysis. Plasma corticosterone, *Crh* (corticotropin-releasing hormone) mRNA expression in the hypothalamus (HT) and C-FOS expression in the paraventricular nucleus of the HT (PVN) in response to an acute stress (10 min of open-field) were examined, as well as hippocampal neurogenesis. Visceral sensitivity was evaluated in a separate set of animals at adulthood (2 months, cohort 3).

The following experiments were conducted in stressed dams fed a SD or HFD (PP2-14, cohort 1). Food intake during the 180 min separation and out of the stress-sessions in the home cage was measured. Maternal care was analyzed on PP2 and PP10 in a subset of stressed dams. At PP11, blood samples were withdrawn at the end of the 180 min of separation for corticosterone determination. Dams' anxiety-like behavior was tested in the light–dark test on the last day of the MS procedure (PP14). Dams were killed 2 weeks after weaning and fat tissue (mesenteric and perigonadic) was collected and weighed.

### Behavioral assessments in adult offspring

#### Open field

Rats were placed in the corner of the open field (100 × 100 cm) and exploration of the center (40 × 40 cm) was recorded for 10 min using videotracking (Bioseb, Vitrolles, France). Distance traveled and number of visits in the center were automatically quantified.

#### Sucrose preference

Rats were individually housed and presented two bottles of tap water to measure basal water consumption. After 48 h of habituation, animals were presented one bottle filled with 1% sucrose solution and one bottle of water. Both intakes were measured after 24 h of test and sucrose preference was calculated as percentage of the volume of sucrose intake over the total volume of fluid intake. Bottle side was randomized to control for any side bias.

#### Social interaction

Pairs of weight-matched rats from the same experimental group were placed in a new cage, under dim light (30 lux) for 8 min. Social behavior (sniffing, allogrooming and crawling over) were recorded and scored using an ethological software (The observer, Noldus Information Technology, Wageningen, The Netherlands).^31^

#### Morris water maze

Spatial learning and memory were assessed as previously described.^28^ Learning consisted of six sessions (four daily trials each) during which distance traveled to reach the hidden platform was recorded (Bioseb, Vitrolles, France). After the last session, animals were given 48 h of retention time and were tested for reference memory during a 90 s probe trial without the platform. Time spent in each quadrant was analyzed.

#### Colorectal distension

Visceral sensitivity was evaluated using electromyography recordings in response to progressive colorectal distension as previously described.^32^ For more information see Supplementary Methods.

### Behavioral assessments in stressed dams

#### Food consumption

From PP2 to PP14, 24 h and 180 min stress food intakes were measured. Data were expressed as a percentage of food intake during separation on MS day 2.

#### Light-dark box

On the last day of separation (PP14), dams' anxiety was assessed in the light–dark box paradigm during separation. The total time spent in the light compartment was recorded for 10 min as previously described.^31^

#### Maternal behavior

On PP2 and PP10, five 60-min periods in the light (1300 and 1900 hours) and dark (2200, 0100 and 0500 hours) phases were video-recorded. Dams' behaviors were scored every 5 min (12 observations per hour) and classified into either ‘maternal behavior' (arched back posture, licking/grooming and passive nursing, including nesting and pup retrieving) or ‘non-maternal behavior' (off nest, including eating/drinking, self-grooming).^33^

### Molecular and biochemical analysis

#### Real-time quantitative PCR (pups and adult offspring)

Total mRNA was extracted from PFC of pups and HT of adult offspring using a TRIzol extraction kit (Invitrogen, Life Technologies, Carlsbad, CA, USA) according to the manufacturer's instructions. The RNA concentration and purity were determined using a ND-1000 spectrophotometer (Nanodrop Technologies, Wilmington, DE, USA). cDNA was synthestized from 1 μg of RNA using Superscript III reverse transcriptase (Invitrogen, Life Technologies) as previously described.^34^ Quantitative PCR was performed using SYBR assays (Supplementary Table 2). See Supplementary Methods for further details.

#### Plasma metabolic hormones multiplex assay (pups)

Trunk blood was collected from PND11 pups after 180 min of MS and centrifuged at 4 °C before plasma was stored at −20 °C. Plasma leptin, insulin, total glucagon-like peptide 1 (GLP-1) and peptide YY (PYY) of PND11 pups were measured by multiplex assay (MILLIPLEX MAP Rat Metabolic Hormone Magnetic Bead, Millipore, Fontenay sous Bois, France) according to the manufacturer's instructions. Hormone concentrations were determined using the Luminex xMap Technology (Bio-Rad, Marnes-la-Coquette, France). All samples were processed in duplicates. Intra and inter assay coefficients were below 15% and crossed reactions were insubstantial (0.01%).

#### Corticosterone radioimmunoassay (dams and adult offspring)

Blood samples were collected from the tail vein (between 0900 and 1200 hours), centrifuged at 4 °C and plasma was stored at −20 °C until use. Total plasma corticosterone was measured with an in-house radio immunoassay, by competition between cold corticosterone (B) and 3H-B (B*) for a specific anticorticosterone antibody, as previously described.^35^ The sensitivity of this assay is around 5 ng ml^−1^. Intra- and interassay variations were <15%.

### Immunohistochemistry in adult offspring

#### Neuronal activation in PVN

Anesthetized rats (Pentobarbital, 50 mg kg^−1^) were intracardially perfused with Phosphate Buffer Solution followed by 4% Paraformaldehyde. Brains were post-fixed in the same fixative for 24 h, cryoprotected in 30% sucrose, and stored at −80 °C until use. Immunostaining for C-FOS was used to measure neuronal activation 1 h post stress. Free-floating sections (40 μm) containing PVN (−1.80 to −2.12 mm posterior to Bregma)^36^ were treated as previously described.^37^ C-FOS immunoreactive (IR) cells were counted with the optical fractionator method using a microscope (Olympus, Hamburg, Germany, BX51) equipped with an objective (× 100), a video camera (Nikon digital camera DMX 1200, Champigny sur Marne, France), and a stereological software (Mercator, ExploraNova, La Rochelle, France). Quantification of C-FOS-IR cells was carried out in two PVN sections per animal. The fields of view were systematically sampled using a step size of 50 μm along the *x* and *y* axes. The dissector counting frames were 150 × 150 μm. Results are expressed as C-FOS-IR cells in the total PVN.

#### Hippocampal neurogenesis

Hippocampal sections (from bregma 2.30 to 5.20 mm) were treated for doublecortin (DCX) immunoreactivity using a goat polyclonal antibody (1:1000, Santa Cruz Biotechnology, Santa Cruz, CA, USA) and a biotinylated donkey anti-goat secondary antibody (1:200, Amersham, Chicago, IL, USA) as previously described.^38^ Adult neurogenesis in the dentate gyrus (DG) was evaluated in eight coronal slices of hippocampus. For each rat (*n*=4 per group), four matched-sections for dorsal (1.06 to −2.06 mm) and ventral (−3.08 to −3.80 mm) hippocampus were selected.^39^ DCX-IR cells were counted within the granular cell layer. The number of DCX-IR cells was then expressed per mm^2^.

### Statistical analysis

Three different cohorts were used to evaluate the independent and combined effects of maternal HFD and MS. Sample sizes were determined based on power analysis and common practice in behavioral (~10 animals per group) and molecular biology (~5 animals per group) experiments. The exact number of animals tested in each group is specified in the figure legends. All data were analyzed using Statistica 6.0 (StatSoft, Tulsa, OK, USA). Graphs showing the means±s.e.m. were graphed using Prism 5.0 (GraphPad Software, San Diego, CA, USA). Normality was assessed by Shapiro–Wilk tests. Statistical outliers were detected with the Grubb's test and highly significant outliers (*P*<0.01) were removed from analyses. Data were analyzed using two or three-way analysis of variance (ANOVA) with repeated measures when appropriate, followed by Fisher's LSD *post hoc* tests or planned comparisons (% dams' food intake throughout the maternal separation sessions). Unpaired Student *t*-tests were used to test the effects of maternal diet in stressed animals. Pearson correlation was used to examine the link between anxiety in the open-field and memory performance in the water maze. Neurogenesis and maternal behavior were analyzed by Mann-Whitney *U*-tests. Data quantifications that potentially include subjective bias (social interaction, maternal care, C-FOS and DCX quantification) were conducted by observers blind to the experimental group. Statistical significance was set at *P*<0.05.

## Results

### Maternal high-fat diet prevents the molecular signature of maternal separation in pup's prefrontal cortex

**Figure 1:**
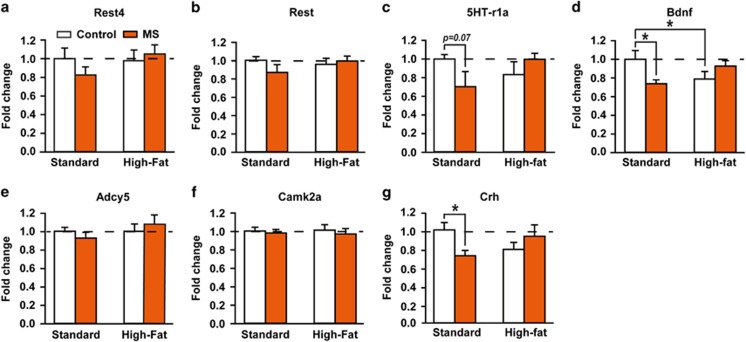
Independent and combined effects of maternal high-fat diet and maternal separation on pups' prefrontal cortex gene expression. Gene expression of (**a**) *Rest4*, (**b**) *Rest*, (**c**) *5HT-r1a*, (**d**) *Bdnf*, (**e**) *Adcy5*, (**f**) *Camk2a* and (**g**) *Crh* in the PFC of 11-day-old pups (*n*=10 for SD–control and HFD–control, *n*=7 for SD–MS and *n*=9 for HFD–MS; except for *Camk2a: n*=5 for SD–MS, *n*=9 for HFD–control and *n*=8 HFD–MS). All data are expressed relative to the housekeeping gene *β2m* (fold change). Pups of SD-fed dams exposed to chronic MS exhibited a slight decrease in *5HT-r1a* mRNA levels, and a significant down-regulation of *Bdnf* and *Crh* mRNA. Expression of these markers was restored by maternal HFD. *Adcy5*, Adenylate cyclase5; *Bdnf*, Brain-derived neurotrophic factor; *Camk2a*, Calcium/calmodulin-dependent protein kinase 2 α *Crh*, Corticotropin-releasing hormone; HFD, high-fat diet; MS, maternal separation; *Rest*, Neural-restrictive silencer element, repressor element 1 (RE1), silencing transcription factor; *Rest4*, *Rest* splicing variant 4; SD, standard diet; *5HT-r1a*, Serotonin receptor 1A. *P<0.05.

To examine the respective and combined effects of maternal HFD and MS on the developing brain, we assessed mRNA expression of *Rest4* and related genes in the PFC of PND11 pups, with stressed pups killed before the stress session (Figure 1a–g). Expression of the housekeeping *B2m* gene did not significantly vary across groups in any of condition (data not shown). *Rest4*, *Rest*, *Adcy5* (adenylate cyclase 5) and *Camk2a* (calcium/calmodulin-dependent protein kinase 2 α) mRNA levels were not significantly altered by maternal HFD nor MS (Figure 1a). However, there was a significant interaction between maternal diet and early stress for *5HT-r1a* (two-way ANOVA, *F*_(1,31)_=4.3221, *P*=0.0460), *Bdnf* (*F*_(1,32)_=6.6418, *P*=0.0148) and *Crh* (*F*_(1,32)_=5.3553, *P*=0.0272) mRNA levels (Figure 1c–g). Pups of SD dams exposed to chronic MS exhibited a trend toward a decrease of *5HT-r1a* mRNA (Fisher's LSD *post hoc,* SD–MS versus SD–control, *P*=0.0726), and a significant downregulation of *Bdnf* (*P*=0.0282) and *Crh* (*P*=0.0443) mRNA expression. The effect of maternal HFD alone was restricted to a decrease of *Bdnf* expression (HF-control versus SD–control, *P*=0.0482). Unexpectedly, the combination of maternal HFD and MS led to *5HT-r1a*, *Bdnf* and *Crh* mRNA levels similar to control levels (HFD–MS versus SD–control, *P*=0.0980, *P*=0.4979, *P*=0.5887, respectively), suggesting a preventive effect of maternal HFD on the developing PFC in MS pups.

**Figure 2:**
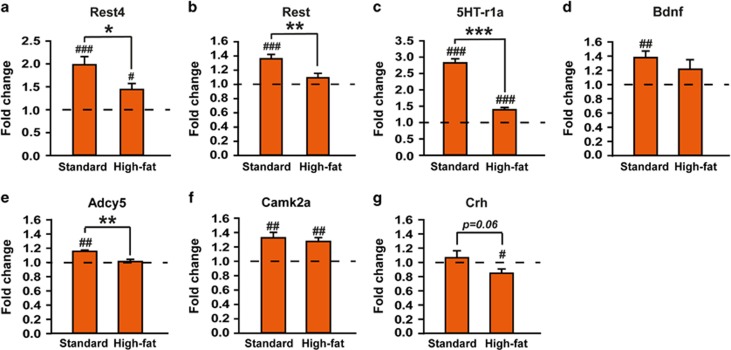
Maternal high-fat diet prevents gene expression changes in the prefrontal cortex of pups following 180- min of maternal separation. mRNA levels of (**a**) *Rest4*, (**b**) *Rest*, (**c**) *5HT-r1a*, (**d**) *Bdnf*, (**e**) *Adcy5*, (**f**) *Camk2a* and (**g**) *Crh* in the PFC of 11-day-old pups after 180-min of separation (*n*=8 per group). All data are expressed relative to gene expression in pups killed before separation. In pups of SD-fed dams, expression of all these markers, except *Crh*, was increased after the acute separation, compared with baseline. However, the acute separation-induced rises of *Rest*, *Rest4*, *5HT-r1a* and *Adcy5* were blunted in pups born to high-fat-fed dams. *Adcy5*, Adenylate cyclase5; *Bdnf*, Brain-derived neurotrophic factor; *Camk2a*, Calcium/calmodulin-dependent protein kinase 2 α *Crh*, Corticotropin-releasing hormone; PFC, prefrontal cortex; *Rest*, Neural-restrictive silencer element, repressor element 1 (RE1), silencing transcription factor; *Rest4*, *Rest* splicing variant 4; SD, standard diet; *5HT-r1a*, Serotonin receptor 1A. *P<0.05, **P<0.01 and ***P<0.001; ^#^*P<*0.05, ^##^*P<*0.01 and ^###^*P<*0.001 compared with the standard value of 1 representing mRNA levels in pups killed before the separation.

On PND11, we further examined PFC gene expression at the end of the stress session (that is, 180-min) in chronically stressed pups, as compared with expression in stressed animals (SD or HFD) killed before stress (Figure 2a and g). There was a significant rise in mRNA expression of *Rest4* (one sample *t*-test, *t*_(7)_=5.5237, *P*=0.0009), *Rest* (*t*_(7)_=6.2155, *P*=0.0004), *5HT-r1a* (*t*_(7)_=14.4684, *P<*0.0001), *Bdnf* (*t*_(7)_=4.2398, *P*=0.0038), *Adcy5* (*t*_(7)_=4.7668, *P*=0.0020) and *CamK2a* (*t*_(7)_=4.3180, *P*=0.0035) in stressed pups of SD-fed dams (Figure 2a and f). Maternal HFD strongly blunted MS-induced upregulation of *Rest4* (unpaired Student *t*-test, *t*_(14)_=2.4514, P=0.0280), *Rest* (*t*_(14)_=3.4289, *P*=0.0041), *5HT-r1a* (*t*_(14)_=9.9536, *P<*0.0001) and *Adcy5* (*t*_(14)_=3.0611, *P*=0.0085), but not *Bdnf* nor *Camk2a* (*t*_(14)_=1.0520, *P*=0.3106 and *t*_(14)_=0.5439, *P*=0.5951, respectively). Finally, *Crh* mRNA levels were decreased in MS pups from dams fed with HFD (one sample *t*-test, *t*_(7)_=2.4416, *P*=0.0581; Figure 2g). Overall, our data highlight an unexpected, protective effect of maternal HFD on the molecular changes associated with MS during brain development.

### Maternal high-fat diet prevents adult endophenotypes associated with early-life stress

**Figure 3:**
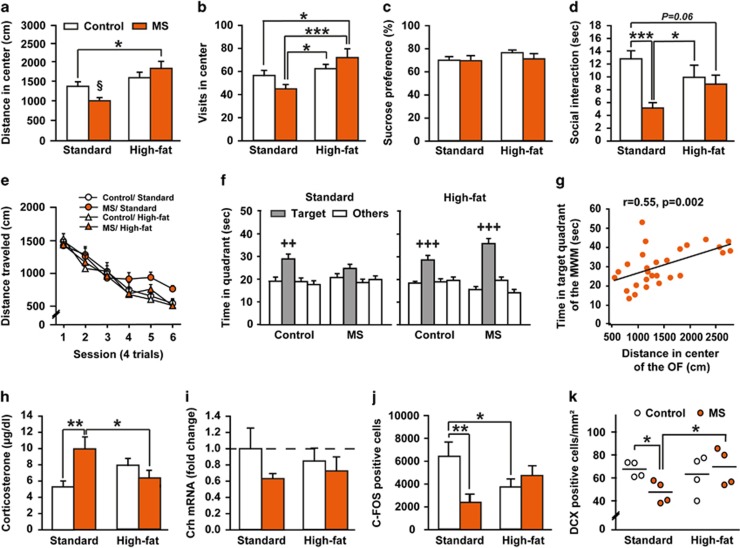
Maternal high-fat diet alleviates offspring endophenotypes induced by maternal separation. (**a**) Distance traveled (cm) and (**b**) number of visits in the center of the open field (*n*=15 for SD-control and HFD–control, *n*=14 for SD–MS and HFD–MS). (**c**) Sucrose preference (percentage of sucrose solution consumption relative to total fluid intake) over 24 h (*n*=15 for SD–control and HFD–control, *n*=14 for SD–MS and HFD–MS). (**d**) Time (sec) spent in social interaction (*n*=7 *per* group). (**e**) Distance traveled (cm) to reach the hidden platform during learning and (**f**) time (sec) spent in the target quadrant during the probe test, 48 h after the last training session (*n*=15 for SD–control and HFD–control, *n*=14 for SD–MS and HFD–MS). (**g**) Significant positive correlation between distance traveled in the center of the open field and time spent in target quadrant in the water maze in adult offspring exposed to MS (*n*=28). (**h**) Plasma corticosterone levels (μg dl^−1^) (*n*=15 for SD–control and HFD–control, *n*=14 for SD–MS and HFD–MS), (**i**) *Crh* mRNA expression in the hypothalamus (fold change) (*n*=6 for SD–control, SD–MS and HFD–MS; *n*=7 for HFD–control) and (**j**) Number of C-FOS-IR cells in the PVN 1 h after 10 min open-field exposure (*n*=8 for SD–control, SD–MS and HFD–control; *n*=7 for HFD–MS). (**k**) Number of DCX-IR cells in the DG of the hippocampus (cells per mm^2^; *n*=4 *per* group). *Crh*, Corticotropin-releasing hormone; DCX, Doublecortin; HFD, high-fat diet; MS, maternal separation; SD, standard diet. *P<0.05, **P<0.01 and ***P<0.001; ^§^*P*=0.07 compared with SD–control and *P<*0.05 compared with HFD–control and HFD–MS; ++ at least *P<*0.01 and +++ at least *P<*0.001 compared with all other quadrants.

Since *Rest4* overexpression in the mPFC during development leads to long-lasting deleterious effects resembling the MS phenotype,^27^ we hypothesized that the restoration of *Rest4* expression in the PFC at PND11 would alleviate MS-associated behavioral endophenotypes in adult offspring of HFD-fed dams. Therefore, we next examined anxiety, anhedonia, social behavior and spatial learning and memory, which have been extensively reported as affected in adult MS offspring^10, 11, 13, 40^ (Figure 3a and f). In the open-field test, the effects of MS on the distance traveled in the center area differed with respect to the maternal diet (two-way ANOVA, maternal diet × early stress effect: *F*_(1,54)_=4.8826, *P*=0.03138; Figure 3a). In offspring of SD dams, MS tended to decrease the distance in center compared with the control group (Fisher's LSD *post hoc,* SD–MS versus SD–control, *P*=0.0676), suggesting a higher anxiety. This effect was attenuated in MS offspring exposed to maternal HFD (HFD–MS versus SD–control, *P*=0.0001). Maternal HFD exposure had no impact on anxiety-like behavior in non stressed animals (HFD–control versus SD–control, *P*=0.23791). A similar profile was found for the number of visits in the center (two-way ANOVA, maternal diet × early stress effect: *F*_(1,54)_=4.3119, *P*=0.0426; Figure 3b). In contrast, anhedonia, assessed by the sucrose preference test was not significantly altered by MS or maternal HFD (maternal diet × early stress effect: *F*_(1,54)_=0.5020, *P*=0.4818; Figure 3c). In the social interaction test, MS rats spent significantly less time in interaction over the 8-min of the test compared with controls, independently of maternal diet (three-way ANOVA with repeated measures, early stress effect: *F*_(1,24)_=6.9739, *P*=0.0143; data not shown). However, the analysis of the first minute, which can be considered the most anxiogenic, revealed a significant interaction between early stress and maternal diet (two-way ANOVA, *F*_(1,24)_=6.1909, *P*=0.0202; Figure 3d). MS rats exposed to a maternal SD displayed reduced social interaction time compared with their control counterparts (Fisher's LSD *post hoc*, SD–MS versus SD–control, *P*=0.0007). In contrast, in offspring of HFD dams, MS had no effect on social behavior (HFD–MS versus HFD–control, *P*=0.7355). Again, maternal HFD alone had no significant impact on social behavior (HFD–control versus SD–control, *P*=0.1139). As early-life stress is also associated with cognitive dysfunctions,^12, 15^ we examined the impact of maternal HFD on spatial memory performance in the water maze task. All groups performed equally over the spatial learning sessions (three-way ANOVA with repeated measures, maternal diet × early stress × session effect: *F*_(5,270)_=0.6710, *P*=0.6459 (Figure 3e). In the probe test 48 h later (Figure 3f), control offspring of SD and HFD dams spent significantly more time in the target quadrant compared with other quadrants (ANOVA with repeated measures, quadrant effect: SD–control, *F*_(3,42)_=6.1010, *P*=0.0015; HFD–control *F*_(3,42)_=8.1473, *P*=0.0002; Figure 3f, left panel). In contrast, offspring of SD-fed dams submitted to MS did not discriminate the target quadrant (quadrant effect: *F*_(3,39)_=1.4501, *P*=0.2431). This memory impairment was suppressed by maternal HFD (quadrant effect: *F*_(3,39)_=25.1083, *P<*0.0001; Figure 3f, right panel). Given that the water maze task is aversive, we tested a possible link between memory performance and anxiety levels. We found a significant positive correlation between distance traveled in the center of the open-field and time spent in target quadrant of the water maze during the probe test in the MS groups (Pearson correlation: *r*=0.55, *P*=0.002; Figure 3g). Indeed, animals that are the most anxious also exhibit the lowest spatial memory performance, suggesting that exacerbated anxiety may be involved in the memory deficit reported in MS animals.

To better characterize the effect of maternal HFD on MS-associated endophenotypes, we next explored HPA axis response to stress (including plasma corticosterone, *Crh* mRNA expression in the HT, and C-FOS expression in the PVN) and hippocampal neurogenesis, both widely reported as affected by early-life stress.^10, 11, 12, 13, 40^ At the end of the behavioral characterization, animals were killed following an acute stress (open-field exposure; Figure 3h and k). The effect of MS on plasma corticosterone levels differed with respect to the dam's diet (two-way ANOVA, maternal diet × early stress effect: *F*_(1,54)_=8.7133, *P*=0.0047). MS offspring of SD dams exhibited significantly higher plasma corticosterone levels compared with controls (Fisher's LSD *post hoc,* SD–MS versus SD–control, *P*=0.0030). Maternal HFD *per se* tended to produce similar effects to MS (HFD–control versus SD–control, *P*=0.0824). In contrast, MS offspring of HFD dams displayed normalized corticosterone levels (HFD–MS versus SD–control, *P*=0.5072) (Figure 3h). Whatever the maternal diet, there was a non-significant decrease of *Crh* gene expression in the hypothalamus of early stressed animals (two-way ANOVA, early stress effect: *F*_(1,21)_=1.9295, *P*=0.1794; Figure 3i). In addition, we stereologically counted C-FOS IR cells in the PVN of the hypothalamus (Figure 3j). Again, there were differential effects of MS depending on the maternal diet (two-way ANOVA, maternal diet × early stress effect: *F*_(1,27)_=7.9363, *P*=0.0090). In comparison with control SD animals, MS or maternal HFD similarly decreased the number of C-FOS-positive cells in the PVN (Fisher's LSD *post hoc,* SD–MS versus SD–control, *P*=0.0034; HFD–control versus SD–control, *P*=0.0397). In contrast, MS offspring of HFD dams showed normalized C-FOS expression (HFD–MS versus SD–control, *P*=0.2148). Finally, neurogenesis was examined by counting the total number of DCX-positive cells in the DG of the hippocampus. In offspring of SD dams, the total number of DCX-positive cells was significantly lowered by MS (Mann-Whitney *U-*test, SD–MS versus SD–control, *U*=0.0000, *P*=0.0286) (Figure 3k). This MS-induced decrease of newborn neurons was not observed in offspring of HFD dams (HFD–MS versus SD–control, *U*=8.0000, *P=1*.0000). Maternal HFD alone did not affect hippocampal neurogenesis in control animals (*U*=8.0000, *P*=1.0000).

**Figure 4:**
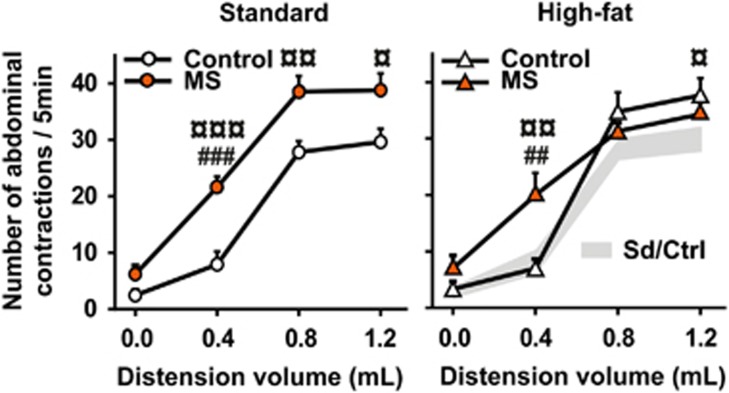
Impact of maternal high-fat diet and maternal separation on offspring visceral sensitivity at adulthood. Number of abdominal contractions (number/5 min) in response to gradual colorectal distension volumes (*n*=11 for SD–control; *n*=10 for SD–MS; *n*=9 for HFD–control and HFD–MS). ^¤^*P<*0.05, ^¤¤^*P<*0.01 and ^¤¤¤^*P<*0.001 compared with control SD; ^##^*P<*0.01 and ^###^*P<*0.001 compared with control HFD. HFD, high-fat diet; MS, maternal separation; SD, standard diet.

In humans, anxiety disorders are highly co-morbid with the IBS, which is characterized by chronic visceral pain.^41^ Since MS is widely used as an animal model of IBS,^14^ we next evaluated visceral sensitivity to pain using colorectal distension.^42^ Gradual colorectal distension increased the number of abdominal contractions in a volume-dependent manner (three-way ANOVA with repeated measures, volume effect: *F*_(3,105)_=176.9673, *P<*0.0001), and this effect was modulated by both maternal diet and early stress (maternal diet × early stress × volume effect: *F*_(3,105)_=2.8488, *P*=0.0410; Figure 4). Specifically, in offspring of SD-fed dams, MS significantly increased abdominal contractions compared with the control group (Fisher's LSD *post hoc,* SD–MS versus SD–control, *P*=0.0011, *P*=0.0089 and *P*=0.0230 for 0.4, 0.8 and 1.2 ml, respectively; Figure 4, left panel). Maternal HFD alone significantly increased the number of abdominal contractions for the distension volume of 1.2 ml (HFD–control versus SD–control, *P*=0.0505). In contrast, maternal HFD suppressed the effect of MS for the highest volumes of 0.8 and 1.2 ml (HFD–MS versus SD–control, *P*=0.3813 and *P*=0.2441, respectively; Figure 4, right panel), without any protective effect on pain threshold (0.4 ml distension volume, *P*=0.0043).

### High-fat diet consumption dampens anxiety and increases maternal care in stressed dams

**Figure 5:**
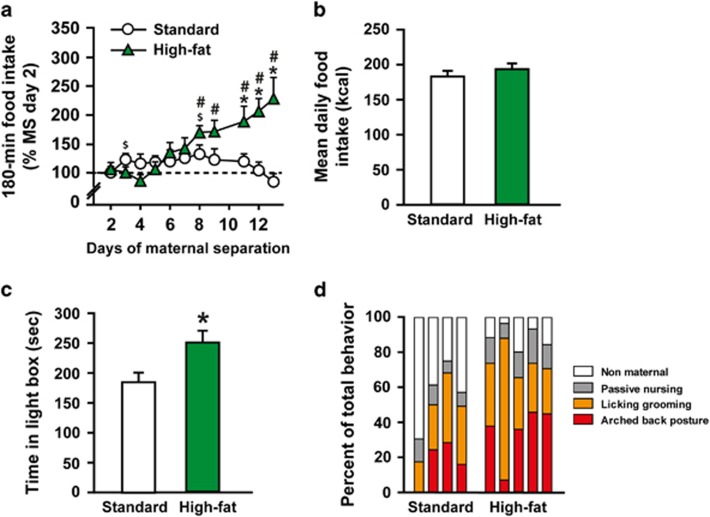
Impact of high-fat diet consumption on stressed dams' behavior. (**a**) Food intake (% of intake on MS-day 2) during the 180 min separations and (**b**) mean daily food intake (kcal) over the two weeks of the MS procedure (*n*=13 for SD–MS and *n*=12 for HFD–MS). Stressed dams fed a HFD increased their food intake during the 180 min of separation over the 2 weeks of MS and consumed significantly more food compared with standard diet (SD)-fed stressed dams. However, daily food intake (homecage) was not different between standard and high fat-fed dams. (**c**) Time (s) in light compartment in the dark/light box on PP14 (*n*=13 for SD–MS and *n*=12 for HFD–MS). HFD dams showed a reduction of their anxiety-like behavior. (**d**) Dams' behavior (percent of total behaviors measured) during the dark phase at PP2 (*n*=4 for SD–MS and *n*=5 for HFD–MS). HFD dams displayed higher global maternal care toward their progeny (arched back posture, licking–grooming and passive nursing together). * at least *P<*0.05 compared with standard diet; $ at least *P<*0.05 compared with MS-day 2 in SD–MS; # at least *P<*0.05 compared with MS day 2 in HFD–MS. HFD, high-fat diet; MS, maternal separation.

Maternal HFD had no impact on dams' body weight at the end of gestation (SD 430.8±11.8, *n*=8; HFD 415.2±11.2 g, *n*=9; *t*_(15)_=0.9555, *P*=0.3545), nor on fat mass at death (2 weeks after weaning; fat mass expressed in % of dams' body weight: SD–MS, 4.11±0.27, *n*=13; HFD–MS, 3.69±0.32, *n*=12; *t*_(23)_=1.0322, *P*=0.3127). Pups' body weight at birth (SD 7.19±0.15, *n*=24; HFD 6.94±0.21 g, *n*=24; *t*_(46)_=0.9532, *P*=0.3455) or after 180-min of MS (Supplementary Table 3) was not affected by maternal HFD either. Moreover, plasma levels of leptin, insulin, GLP-1 and PYY in stressed animals were not significantly different between SD and HFD pups (Supplementary Table 3), suggesting that the protective effect of maternal HFD could not be explained by metabolic adaptations. Considering that separation from pups constitutes a potent stress for dams^43^ and that HFD consumption could exert an anti-stress effect in MS dams, we thus explored the effects of HFD consumption in stressed dams (Figure 5). Consistently, HFD dams increased their food intake during the 180-min MS sessions over the 2 weeks compared with SD dams (two-way ANOVA with repeated measures, maternal diet × separation day effect: *F*_(10,230)_=8.8712, *P<*0.0001; planned comparisons, SD–MS versus HFD–MS, at least *P<*0.05 for sessions 11, 12 and 13; Figure 5a). However, mean daily energy intake during the 2 weeks of MS was similar between SD and HFD dams (unpaired Student *t*-test, *t*_(23)_=0.7684, *P*=0.4501) (Figure 5b), suggesting that HFD dams efficiently adapted their intake with respect to the calories provided by the HFD. We also found a significant reduction of anxiety levels in stressed dams fed a HFD (Figure 5c). Indeed, stressed HFD dams spent more time in the light compartment of the light-dark box compared with stressed SD dams (unpaired Student *t*-test, *t*_(23)_=2.5346, *P*=0.0185). However, changes in anxiety were not associated with significant differences in plasma corticosterone levels after stress (PP11, SD 14.4±2.1, *n*=14; HFD 12.9±1.4 μg dl^−1^, *n*=11; *t*_(23)_=0.5826, *P*=0.5659). We finally explored the impact of HFD on maternal care, which is determinant for later vulnerability to stress^44^ and has been recently demonstrated to be increased in HFD-fed dams.^45^ Stressed dams under HFD displayed increased global maternal care (that is, arched back posture, licking–grooming, and passing nursing) during the dark phase at PP2 (Mann–Whitney *U*-test, *U*=0.0000, *P*=0.0357; Figure 5d). This effect was no longer present on PP10 (data not shown). Of note, maternal care during the light phase was not significantly affected by maternal diet (data not shown).

## Discussion

Early-life stress is associated with increased vulnerability to neuropsychiatric diseases later in life.^1, 2, 5, 6^ Similarly, obesity, excessive weight gain, metabolic disorders and unhealthy HFD during pregnancy have been recently hypothesized to increase the incidence of mental health disorders.^7^ Here we examine whether maternal HFD can have similar effects to MS and/or can exacerbate the effects of MS in the offspring. Contrary to our hypotheses, maternal HFD alone has only small impact on gene expression in pups' PFC and on behavior in adulthood. More importantly, maternal HFD alleviates MS-induced endophenotypes (anxiety, spatial memory, social behavior, HPA axis response to stress and visceral pain) in adulthood and reduces maternal anxiety in stressed dams.

HFD exposure has been recently proposed to act as a stressful challenge during pregnancy.^46^ Herein, we report that maternal HFD (40% from fat) alone has no major consequence in offspring, neither on PFC gene expression in pups nor on behavior in adults. Indeed, as we previously reported,^28^ HFD (40% fat) exposure restricted to gestation and lactation did not lead to maternal overweight. In contrast, in studies where the dams are overweight or obese (60% fat or longer HFD exposure), maternal HFD could impact the developing brain^19, 47^ and lead to exacerbated anxiety^21, 22, 23^ impaired memory^20^ and decreased neurogenesis^19^ in adult offspring. Taken together, these results suggest that maternal obesity, rather than maternal HFD consumption, is critical for the detrimental effects of HFD in offspring.

A major finding of the present study is that early exposure to HFD unexpectedly prevents neurodevelopmental gene expression alterations in the PFC of stressed pups. Indeed, chronic MS led to downregulated *5HT-r1a*, *Crh* and *Bdnf* baseline mRNA levels in PND11 pups, which is prevented by maternal HFD exposure. Interestingly, BDNF and serotonin, notably through the 5HT-R1A receptors, are necessary for proper wiring of neural circuits during development,^48, 49, 50, 51, 52^ shaping normal anxiety in adulthood.^53^ An ontogenic upregulation of *Rest4* and associated markers has been previously reported in the mPFC of MS pups, but not in the amygdala or in the hippocampus.^27^ Here we demonstrate that maternal HFD reversed the MS-induced upregulation of *Rest4* and related genes following separation. As *Rest4* overexpression in the mPFC during development leads to hyperanxiety in adulthood,^27^ our data suggest that the protective effects of maternal HFD exposure on behavior in adulthood might in part result from the normalization of *Rest4* mRNA levels in pups' PFC. We do not rule out that MS-induced changes in gene expression during development also take place in other brain structures. Furthermore, alterations occurring in the PFC during the perinatal period may lead to altered connectivity with other brain areas such as the amygdala or the hippocampus, which have been shown to play a role in anxiety and cognitive functions.

Although the molecular mechanisms underlying the protective effects of maternal HFD remain to be elucidated, our results demonstrate that HFD exposure early in life attenuates MS-induced hyperanxiety, but also MS-related impairments in spatial memory, social behavior and visceral pain. Moreover, hypercorticosteronemia and altered hippocampal neurogenesis, which are associated with hyperanxiety and spatial memory disturbances,^54, 55^ were ameliorated by maternal HFD exposure in MS animals. Previous studies have shown that palatable food consumption in adulthood can attenuate the deleterious effects of early stress on emotional behaviours.^11, 56, 57, 58^ However, to our knowledge, our work is the first to demonstrate that an exposure to HFD restricted to the developmental period can protect against the long lasting disturbances induced by early stress.

The protective effects of maternal HFD on the offspring might depend upon several mechanisms acting synergistically. In particular, it could affect pups' metabolism through feeding or pups' stimulation through the level of maternal care.^59^ In stressed pups, neither body weight nor plasma metabolic markers after the 180 min separation differed according to the maternal diet. Thus, maternal HFD does not lead to a better metabolic adaptation to the 3 h fasting occurring during the separation sessions. A large body of evidence highlights the importance of maternal behavior in later offspring emotional behavior and HPA response to stress.^60^ Moreover, previous work reports that dams maintained on HFD during lactation spent more time nursing their pups.^45^ Consistently, we showed that stressed dams fed a HFD increased care toward their pups compared with stressed SD dams. In humans, it has been demonstrated that food choices are modified under stress with a shift in preference toward more palatable, energy-dense snacks.^61^ Dallman proposed that overconsumption of palatable food dampens negative emotions associated with stress.^62^ MS acts as a potent stressor for dams.^43, 63^ We demonstrate that stressed dams fed a HFD increased their food intake specifically during the 180-min stress session of separation, an effect associated with a reduction of their anxiety. These results extend previous findings showing that high-fat intake modulates stress response in adult animals^11, 64, 65^ and alleviates postpartum anxiety and depressive-like behavior in mother rats subjected to MS.^66^ Maternal stress is detrimental for maternal care quality,^67^ thus it could be hypothesized that the increase of HFD intake during lactation exerts an anti-stress effect on dams, which could promote maternal behavior allowing optimal brain maturation in pups. Further experiments are needed to confirm the comfort food effect of HFD in stressed dams. As stress increases C-FOS expression, future studies should be conducted to examine whether maternal HFD would blunt dams' C-FOS response to the separation stress. Epigenetic regulations are likely candidate for persistent changes in brain function as a consequence of perinatal environment. Indeed, some of the effects of parental obesity persist across multiple generations.^68^ Therefore, it would be interesting to examine whether the protective effect of maternal HFD could be epigenetically transmitted across generations in our model.

Contrary to the prevailing belief that HFD exposure is detrimental for the developing brain, our results suggest that obesity, rather than fat consumption *per se*, is critical for brain vulnerability. Furthermore, to our knowledge, we report for the first time a protective effect of maternal HFD in a context of early-life stress. Further work is needed to better document and understand this phenomenon. Although maternal HFD prevents stress-induced emotional alterations in our study, it is important to consider other health outcomes, such as effects on metabolic or cardiovascular diseases vulnerability that might be exacerbated by HFD. Overall, our findings highlight the importance of taking nutrition into account in clinical studies on early-life adversity and mental health.
