# Supplementary material for: Maternal high-fat diet prevents developmental programming by early-life stress
Source: Transl Psychiatry. 2016 Nov 29;6(11):e966–. doi: 10.1038/tp.2016.235 (PMC5290357; doi:10.1038/tp.2016.235)
Supplement: Supplementary Information [file tp2016235x1.docx]

**SUPPLEMENTARY INFORMATION**

**SUPPLEMENTARY METHODS**

*Colorectal distension.* Briefly, under general anesthesia (intraperitoneal administration of acepromazine (0.4 mg/kg) (Calmivet, Vetoquinol, Lure, France) and ketamine (75 mg/kg) (Imalgene 1000, Rhône-Merieux, Lyon, France) animals were equipped with three groups of NiCr wire electrodes implanted into the abdominal external oblique muscle and the myoelectrical activity was recorded by an electromyograph at least 5 days after the surgery. Rats were placed in a polypropylene tunnel, and a balloon consisting of an arterial embolectomy catheter (Fogarty; Edwards Laboratories Inc., Santa Ana, CA, USA) was slowly placed 1 cm into the rectum and taped at the base of the tail. The balloon was progressively inflated by steps of 0.4 mL, from 0 to 1.2 mL, each step lasting 5 min. The number of spike bursts (abdominal contractions), registered for each colorectal distension volume applied, was used as a visceral sensitivity index.

*Real-time quantitative PCR.* A mix containing 5µl of cDNA (0.5ng/µl), 10 µL of Mesagreen QPCR MasterMix Low ROX (Eurogentec, Saraing, Belgium) and 2 µL of the primers mix at optimal concentration were used in a total volume of 20 µL. PCR program consisted of the following steps: 95°C for 15min, 45 cycles of 95°C for 15s and 60°C for 30s. The primers for *Rest* and *Rest4* mRNA were originally designed by Uchida and colleagues^27^ and the others were designed with the Primer Express Software (Life Technologies, Applied Biosystems, PE Corporation, NY). All primers used were validated for selectivity and amplification efficiency (primer sequences are listed in Supplementary Table 1). Real time quantitative PCRs were performed using the LightCycler 480 system (LC480, Roche diagnostics, Mannheim, Germany). All samples were measured in duplicates. For post PCR analysis, target gene expression was calculated by the delta-delta Ct method^28^ and normalized to the housekeeping gene beta-2 microglobulin (β2m). All gene expression results are expressed relative to expression in the control group (control offspring of standard-fed dams) which is normalized to the value of 1 (fold change). To evaluate gene expression levels in pups’ PFC after 180min of separation, fold change of each animal was expressed relative to the expression of the corresponding group in pups killed before separation.

**SUPPLEMENTARY TABLES**

**Supplementary Table 1.** Composition of standard and high-fat diets

^1^ Composition of vitamin supplements (g/kg vitamins): sucrose, 549.45; retinyl acetate, 1; cholecalciferol, 0.25; dl-α-tocopheryl acetate, 20; phylloquinone, 0.1; thiamin HCl, 1; riboflavin, 1; nicotinic acid, 5; calcium pantothenate, 2.5; pyridoxine HCl, 1; biotin, 1; folic acid, 0.2; cyanocobalamin, 2.5; choline HCl, 200; DL-methionine, 200, para-amino-benzoic acid, 5; inositol, 10.

^2^ Composition of the mineral mixture (g/kg minerals) : sucrose, 110.7; CaCO_3_, 240; K_2_HPO_4_, 215; CaHPO_4_, 215; MgSO_4_,7H_2_O, 100; NaCl, 60; MgO, 40; FeSO_4_, 7H_2_O, 8; ZnSO_4_, 7H_2_O, 7; MnSO_4_, H_2_O, 1; Na_2_SiO_7_,3 H_2_0, 0.5; AlK(SO_4_)_2_,12 H_2_O, 0.2; K_2_CrO_4_, 0.15, NaF, 0.005, NiSO_4_, 6H_2_O, 0.1; H_2_BO_3_, 0.1; CoSO_4_, 7H_2_O, 0.05; KlO_3_, 0.04; (NH_4_)_6_Mo_7_O_24_, 4H_2_O, 0.02, LiCl, 0.015, Na_2_SeO_3_, 0.015, NH_4_VO_3_, 0.01

Abbreviations: PUFA, Poly unsaturated fatty acids.

**Supplementary Table 2:** Primer pairs used for quantitative RT-PCR assays.

Abbreviations: *Adcy5*, Adenylate cyclase5; *B2m*, β2-microglobulin; *Bdnf*, Brain-derived neurotrophic factor; *Camk2a*, Calcium/calmodulin-dependent protein kinase 2α; *Crh*, Corticotropin-releasing hormone; *HTr1a*, 5-hydroxytryptamine (serotonin) receptor 1a; *Rest*, Neural-restrictive silencer element, repressor element 1 (RE1), silencing transcription factor; *Rest4*, *Rest* splicing variant 4.

**Supplementary Table 3:** Maternal high-fat diet has no effect on body weight and metabolism in pups exposed to maternal separation.

Pups’ body weight (g) after 180-min of separation at PND2 (n=10 and 11 for SD-MS and HFD-MS, respectively) and PND11 (n=8 and 6 for SD-MS and HFD-MS, respectively); plasma levels (pg/mL) of leptin, insulin, total GLP-1 and PYY in PND11 pups after 180-min of separation (n=6/group except for insulin: n=5 and 6 for SD-MS and HFD-MS, respectively).

Abbreviations: GLP-1, Glucagon-like peptide 1; PYY, Peptide YY.

**SUPPLEMENTARY FIGURES**

**Supplementary Figure S1**

**Supplementary Figure S1:** Experimental design**.** Dams (n=76) were fed a standard diet (12% kcal from fat) or a high-fat diet (40%kcal from fat) during gestation and lactation. Pups underwent maternal separation (MS) 3h/day between PND2 and PND14, while controls remained undisturbed. At PND21, pups of the 4 groups (SD-control, HFD-control, SD-MS and HFD-MS) were weaned onto laboratory chow. 3 different cohorts were used for analyses in pups and dams (PND2-PND14, cohort 1) or adult offspring (cohorts 2 and 3).

A set of male pups (cohort 1) was killed on PND11 (either before or after the 180min-period of MS) to assess gene expression in the PFC. In stressed dams, food intake (PP2-14), maternal care (PP2 and PP10), and anxiety-like behavior (PP14) were evaluated. At PP11, blood samples in stressed dams were withdrawn at the end of the 180 min of separation for corticosterone determination.

Adult male rats (4-8 months) were tested for behavioral and neuroendocrine phenotypes (cohort 2). Before sacrifice (8 months), rats were exposed to an acute stress exposure (10 min OF) and blood samples for corticosterone assessment were collected after 1 h of recovery. Half of the animals were used to assess *Crh* gene expression in the hypothalamus by qPCR, and half for immunohistochemistry of C-FOS in the PVN and DCX in the DG of the hippocampus. Colorectal distension was carried out in an additional batch of animals (2 months) to evaluate visceral sensitivity (cohort 3).

Abbreviations : G, Gestational day; PND, Post natal day; MS, Maternal separation; PFC, Prefrontal cortex; OF, Open field; MWM, Morris water maze; HPA, Hypothalamic-pituitary-adrenal; Crh, Corticotropin-releasing hormone; PVN, Paraventricular nucleus of the hypothalamus.
